# Open-Source FPGA Coprocessor for the Doppler Emulation of Moving Fluids

**DOI:** 10.3390/mi12121549

**Published:** 2021-12-12

**Authors:** Stefano Ricci

**Affiliations:** Information Engineering Department, University of Florence, 50139 Florence, Italy; stefano.ricci@unifi.it

**Keywords:** embedded coprocessor, electronic doppler phantom, FPGA, VHDL, doppler ultrasound

## Abstract

Embedded systems are nowadays employed in a wide range of application, and their capability to implement calculation-intensive algorithms is growing quickly and constantly. This result is obtained by the exploitation of powerful embedded processors that are often connected to coprocessors optimized for a particular application. This work presents an open-source coprocessor dedicated to the real-time generation of a synthetic signal that mimics the echoes produced by a moving fluid when investigated by ultrasounds. The coprocessor is implemented in a Field Programmable Gate Array (FPGA) device and integrated in an embedded system. The system can replace the complex and inaccurate flow-rigs employed in laboratorial tests of Doppler ultrasound systems and methods. This paper details the coprocessor and its standard interfaces, and shows how it can be integrated in the wider architecture of an embedded system. Experiments showed its capability to emulate a fluid flowing in a pipe when investigated by an echographic Doppler system.

## 1. Introduction

Advances in fields such as the Internet of Things (IoT), Artificial Intelligence (AI), wearable sensors, industry 4.0, cyber-physical systems, biomedical devices, and many others require electronic devices and systems with higher and higher processing capabilities. Smart embedded processors work in most applications, but in some cases, especially when strict real-time constraints must be satisfied, they do not grant sufficient performance. In these occasions, a fast, specialized coprocessor can represent the right solution.

A coprocessor is often optimized and specialized for a specific problem, and it is required to process a wide amount of data in little time. Field Programmable Gate Arrays (FPGAs) offer all the features needed in the implementation of coprocessors: they allow the realization of specialized parallel architectures and they feature abundant calculation resources. Several examples of coprocessors implemented in FPGAs are indeed already present in literature. They are employed, for example, to help in population count [1], power quality monitoring [2], modular arithmetic [3], object classification [4], computer vision [5], high precision integer arithmetic [6], Convolutional Neural Networks (CNNs) [7,8], real-time signal processing [9], cryptography [10,11], data compression/decompression [12], etc. Moreover, the modern System-on-Chip (SoC) FPGAs include a hard processor as well; thus, the processor plus dedicated coprocessor couple can be efficiently integrated in a single device.

The analysis of flowing fluids through Doppler ultrasound is nowadays performed both in biomedical and industrial applications. For example, medical doctors employ echographs in everyday clinical practice to investigate how blood flows in arteries and veins. In this case, blood velocity is a parameter of particular interest, since it is related to the presence of atherosclerotic plaques [13]. In the industrial field, ultrasound Doppler investigation of flowing fluids is performed, for example, for accurate volumetric flow measurement or for the characterization of fluid rheology [14]. This is achieved, in particular, through the measurement of the velocity distribution that a fluid develops across the diameter of a pipe [15]. Academic and industrial research is quite active in the field, and novel methods and electronic systems are being investigated and designed. Laboratorial tests of such methods and systems are nowadays performed by employing cumbersome and expensive flow-rigs [16] or string phantoms [17], in the effort to reproduce flowing conditions with known characteristics. For example, the accuracy of a Doppler system is tested by investigating a flow established in a flow-rig, and comparing the velocity measured by the system to the ground-truth velocity theoretically present in the flow-rig. Unfortunately, these set-ups are affected by mechanical and fluid dynamic uncertainties that do not allow the exact knowledge of the ground-truth velocity. Moreover, reproducing a known and desired velocity distribution along the diameter (velocity profile) in a flow-rig [18] is quite challenging.

Electronics Doppler Phantoms (EDPs) [19,20,21] represent an interesting solution to this problem. EDPs produce a synthetic signal that emulates the echo that a real flow would have produced. The Doppler system being tested receives such a synthetic signal and processes it as if it originated from a real flowing fluid. Signal features such as velocity profile, ultrasound beam width, wall clutter, etc. can be easily controlled by synthesizing the signal with a suitable mathematical model [21]. Unfortunately, this signal model requires an intensive calculation effort, especially when the signal is generated in real-time. FPGA-based coprocessors can play an important role in this field.

In this paper an open-source FPGA-based coprocessor for the generation of a synthetic Doppler signal is presented. The proposed coprocessor is implemented in a SoC FPGA, integrated in an electronics system based on an FPGA developing board connected to a custom board, and employed as a flexible EDP. Compared to the state-of-the-art, the proposed coprocessor allows, for the first time, the emulation of a flow in a pipe with a programmable velocity distribution along the pipe diameter. While the EDP board has already been detailed in [21], this work focuses specifically on the coprocessor.

The paper proceeds with a brief description of the signal model (see Section 2). Section 3 details the implementation of the coprocessor and its integration with the SoC FPGA for realizing the EDP. Section 4 analyzes the performance and shows the results of a test performed by connecting the EDP to a research echograph. Finally, Section 5 discusses the results and concludes the paper. Appendix A reports, for reader convenience, the main acronyms employed in the paper.

## 2. The Fluid Model

This section includes a brief description of the Doppler signal model implemented in the coprocessor. General detail about the processing of ultrasound Doppler signals can be found in [22,23]; more details about the model are available in [21].

Let us consider a moving fluid as composed by a collection of a multitude of particles that, when lighted by an ultrasound wave, scatter the wave in every direction. Each particle *n*, hereafter named ‘scatterer’, moves along a flowline with velocity $v_{n}$. During an ultrasound inspection, the moving fluid is investigated by transmitting ultrasound bursts at frequency $F_{t}$, which are repeated every Pulse Repetition Interval (PRI) $T_{pri}$. The echo produced by the scatterers is sampled at each PRI at the frequency $F_{t}$ = $1/T_{c}$. If k is the sample index along the depth (typically referred to as ‘fast time’), and l is the sample index along the PRI sequence (typically referred as ‘slow time’), the received echo is represented by a 2D matrix of indexes (k,l) [24]:

(1) $$\begin{array}{c} S_{n}\left(k,l\right)=A_{n}\cdot W_{n}\left(k,l\right)\cdot \sin\left[2\pi \cdot F_{t}\left(k\cdot T_{c}+\frac{l\cdot v_{n}\cdot T_{pri}}{c}\right)\right] \end{array}$$

where *c* is the sound velocity and $A_{n}$ represents the backscattering coefficient. $W_{n}\left(k,l\right)$ is a 2D Blackman tapering window, which determines the extension $K_{n}$, $L_{n}$ of the matrix along the k,l indexes ($0\leq k<K_{n},0\leq l<L_{n}$).

The $K_{n}$ parameter is directly related to the axial Doppler Sample Volume (SV) [22]:

(2) $$K_{n}=\frac{2\cdot SV_{n}}{c\cdot T_{c}}\frac{1}{\alpha }$$

Here $SV_{n}$ is the −6 dB extension of the SV, and $cT_{c}/2$ is the extension of the fast-time sample, i.e., the distance that ultrasound travels back and forth in the sampling time $T_{c}.$ The coefficient α represents the window relative to the −6 dB extension and it counts 0.8 for the Blackman window. Similarly, the parameter $L_{n}$ accounts for the echo extension in the slow-time direction, which is related to the width of the ultrasound beam $BW_{n}$:

(3) $$L_{n}=\frac{BW_{n}}{\left|v_{n}\right|\cdot T_{pri}}\frac{1}{\alpha }$$

Here $BW_{n}/|v_{n}|$ represents the transit time, i.e., the time needed by the scatterer to cross the beam width [25].

The final signal matrix M(k,l) is theoretically composed by summing up the contribution of the collection of scatterers that compose the fluid. For practical reasons, we use *N* scatterers placed in the random positions $\left(k_{n},l_{n}\right)$:

(4) $$M\left(k,l\right)=\overset{N-1}{\underset{n=0}{\sum }}S_{n}\left[k-\left(k_{n}-\frac{K_{n}}{2}\right),l-\left(l_{n}-\frac{L_{n}}{2}\right)\right]$$

In summary, this matrix represents the radio-frequency signal received by a hypothetical transducer that investigates a flow composed by *N* scatterers moving at velocity $v_{n}$. An average of about 10 scatterers in the Doppler sample volume is typically enough to generate a signal with real-like statistic attributes. The desired velocity profile of the flow is emulated by suitably choosing the scatterers positions and velocities. For example, the typical parabolic profile developed by a Newtonian fluid that flows in a pipe is emulated by placing the scatterers with the maximum velocity at the pipe center, and by scaling the lateral scatterers velocity by applying the parabola formula according to their distance from the center.

## 3. FPGA Implementation of the Scatterer Generator

### 3.1. Preliminaries

As stated above, the echo produced by a scatterer is expressed by Equation (1). However, in order to facilitate the hardware implementation of the equation, it is convenient to rearrange the formula in the following form:

(5) $$S_{n}\left(k,l\right)=A_{n}\cdot W_{n}\left(k\right)\cdot W_{n}\left(l\right)\cdot \sin\left[2\pi \left(B_{n}\cdot k+C_{n}\cdot V_{rn}\cdot l\right)\right]$$

The values of the constants are:

(6) $$B_{n}=F_{t}\cdot T_{c};\;\;\;\;\;C_{n}=\frac{F_{t}\cdot T_{pri}}{c}V_{M};\;\;\;V_{rn}=\frac{v_{n}}{V_{M}}\;\;$$

The velocity of the scatterer, which in Equation (1) is $v_{n}$, in the rearranged formulas is expressed through the ratio $V_{rn},$ where the velocity is normalized with respect to the velocity $V_{M}$. The velocity $V_{M}$ can be arbitrary selected; however, the maximum velocity present in the velocity profile, typically localized in the vessel center, is a natural choice. The velocity $V_{M}$ is assumed to be positive; while $V_{rn}$ is positive or negative to account for the scatterer velocity direction, but its absolute value is assumed to be less than 1 ($V_{M}$ > $\left|v_{n}\right|$).

A useful property that arises from the periodicity of the sin function is the following:

(7) sin[2π(x)]=sin[2π(x±N)]

In Equation (7) *N* is an integer number. Adding and/or removing an integer value from the argument of the sin[2π(x)] function does not affect the result. We will use this feature to simplify the data path in the hardware by removing the integer part of the mathematical representation from the data that compose the argument of the function, as detailed in the following sections of this paper.

Equation (5) needs the calculation of the Blackman window $W_{n}\left(x\right)$ and the sinusoidal function sin[2π(x)]. These functions cannot be generated efficiently in-line through additions and multiplications, but they can be easily obtained through look-up tables implemented in the FPGA memories. From Equation (2). we know that the extension of the Blackman window along the *k* index is $K_{n}$. Thus, if the window is tabled in *N* words, the address inside the table as a function of the index *k* is:

(8) $$\mathrm{Add}\left(k\right)=\mathrm{int}\left(\frac{k}{K_{n}}\cdot N\right)=\mathrm{int}\left(k\cdot E_{n}\cdot N\right)\;\;\;\;\;\;\;\;\;\;\;\;\;\;\;\;\;\;\;\;\;\;E_{n}=\frac{c\cdot T_{c}}{2\cdot SV_{n}}\alpha =\frac{1}{K_{n}}\;\;\;\;$$

The int(∙) function returns the integer part of the argument. A similar reasoning can be applied, starting from Equation (3), to the Blackman window along the *l* index. In this case we conclude:

(9) $$\mathrm{Add}\left(l\right)=\mathrm{int}\left(\frac{l}{L_{n}}\cdot N\right)=\mathrm{int}\left(l\cdot D_{n}\cdot N\right)\;\;\;\;\;\;\;\;\;\;\;\;\;\;D_{n}=\frac{V_{M}\cdot V_{rn}\cdot T_{pri}}{BW_{n}}\alpha =\frac{1}{L_{n}}$$

Memory can be saved by exploiting the symmetry of the Blackman window around the central axis. The memory stores the first N/2 samples of the window and the address generation is modified as:

(10) $$\begin{array}{c} \mathrm{Add}\left(l\right)=\{\begin{array}{cc} \mathrm{int}\left(l\cdot D_{n}\cdot N\right), & 0\leq l\cdot D_{n}<0.5\\ N-\mathrm{int}\left(l\cdot D_{n}\cdot N\right), & 0.5\leq l\cdot D_{n}<1 \end{array}\\ \mathrm{Add}\left(k\right)=\{\begin{array}{cc} \mathrm{int}\left(k\cdot E_{n}\cdot N\right), & 0\leq k\cdot E_{n}<0.5\\ N-\mathrm{int}\left(k\cdot E_{n}\cdot N\right), & 0.5\leq k\cdot E_{n}<1 \end{array} \end{array}$$

Since the internal memories of the FPGA are dual port memories, it is possible to simultaneously produce samples of both the Blackman windows by accessing the memory from the two ports.

A similar strategy to save memory is applied to table the sin[2π(x)] function. In this case, memory requirements are reduced four-fold by storing only the first quadrant of the function. However, this time, it is necessary to invert the sign of the sample that is output from the memory when it appeares in the third and fourth quadrant, as detailed below:

(11) $$\begin{array}{c} \mathrm{Add}\left(x\right)=\{\begin{array}{c} \begin{array}{cc} \mathrm{int}\left(x\cdot N\right), & 0\leq x<0.25\\ N-\mathrm{int}\left(x\cdot N\right), & 0.25\leq x<0.5 \end{array}\\ \begin{array}{cc} \mathrm{int}\left(x\cdot N\right), & 0.5\leq x<0.75\\ N-\mathrm{int}\left(x\cdot N\right), & 0.75\leq x<1 \end{array} \end{array}\\ r\left(x\right)=\{\begin{array}{c} \begin{array}{cc} \mathrm{MEM}\left[\mathrm{Add}\left(x\right)\right], & 0\leq x<0.25\\ \mathrm{MEM}\left[\mathrm{Add}\left(x\right)\right], & 0.25\leq x<0.5 \end{array}\\ \begin{array}{cc} -\mathrm{MEM}\left[\mathrm{Add}\left(x\right)\right], & 0.5\leq x<0.75\\ -\mathrm{MEM}\left[\mathrm{Add}\left(x\right)\right], & 0.75\leq x<1 \end{array} \end{array} \end{array}$$

Here r(x) is the final sample; MEM[Add(x)] is the sample present in the table at address Add(x); x is the argument of sin[2π(x)] that corresponds to $B_{n}\cdot k+C_{n}\cdot V_{rn}\cdot l$ (see Equation (5)). Please note that, since the memory integrated in the FPGA has a two-stage pipeline and the sign inversion is applied to the memory data available at the output, the sign bit should follow a parallel pipeline to reach the memory output in sync with the data (see next section).

In this work, we employed 512-word tables and thus we achieved a *N* = 1024 word resolution for the Blackman window and *N* = 2048 word resolution for the sin function.

### 3.2. Dynamics of the Input Parameters

The accelerator should perform the calculations summarized in Equation (5). Its inputs are: $A_{n}$, $B_{n}$, $C_{n}$, $V_{n}$, *l*, and *k*. In addition to these, $D_{n}$ and $E_{n}$ are considered as further inputs, since they are needed in the calculation of the addresses to the Blackman and sin tables as detailed by Equations (10) and (11). The values of $A_{n}$, $B_{n}$, $C_{n}$, $D_{n}$, $E_{n},\;\mathrm{and}$ $V_{n}$ are characteristic of each scatterer, do not change during the calculation of its samples, and thus are pre-calculated. On the other hand, the indexes *l* and *k,* which specify the position of the sample that is currently generated, update their values during the calculation of the scatterer data.

All of these values should be represented in signed or unsigned fixed-point mathematical format. The number of bits employed in their representation is a trade-off between the mathematical resolution and the hardware requirements. In the following section, the suitable representation for each variable will be established. The mathematical format is synthetically represented by the notation (U/S X.Y), where U or S stands for unsigned or signed, respectively, and X,Y are the numbers of bits on the left and right of the fractional point, respectively. 

The parameter *k* is a natural number that ranges from 0 up to $K_{n}$ − 1. $K_{n}$ represents the number of samples that the $SV_{n}$ counts along the direction of the *k* index. Since the depth that a typical system collects to is in the order of hundreds [15], a conservative format for *k* is U10.0. With similar reasoning, we conclude that U10.0 is a suitable representation for the *l* index as well. The velocity ratio $V_{rn}$ is a signed value lower than 1 (see Equation (6)). Doppler ultrasound measurements achieve a typical accuracy in the order of 5–10% [22]; thus S1.10, which allows a resolution of 1/1000, is a suitable representation for $V_{rn}$. The ratio between the transmission and the sampling frequency, $B_{n}$, should be less than 0.5 to be compliant with the sampling frequency limit. It is represented by the U0.16 fractional format. The evaluation of the dynamics of the parameter $C_{n}$ is more complicated. We investigate its range of variation through some extreme examples. According to Equation (6), $C_{n}$ is maximized for high transmission frequencies, high flow velocities, low PRF, and low sound velocity. If we consider, for example, $F_{t}=15\;\mathrm{MHz}$, $V_{M}$ = 10 m/s, $T_{pri}$ =1/100 Hz, and c = 300 m/s, we obtain $C_{n}$ = 5000 s/m; on the contrary, minimizing $C_{n}$, for example, with $F_{t}=0.1\;\mathrm{MHz}$, $V_{M}$ = 10 mm/s, $T_{pri}$ =1/10 kHz, and c = 2000 m/s, obtains $C_{n}$ = 5∙10^−5^ s/m. Based on the variation range 5∙10^−5^–5∙10^+3^ s/m found in these radical examples, we can safely represent $C_{n}$ with the format U10.14. $E_{n}$ and $D_{n}$ are the reciprocals of $K_{n}$ and $L_{n}$; see Equations (8) and (9). Since these are represented with U10.0, a suitable representation for $E_{n}$ and $D_{n}$ could be U0.10. However, since they are involved in further mathematical steps, it is convenient to maintain more fractional digits, and thus we employ U0.16. Finally, the scattering amplitude $A_{n}$ multiplies the values of the sin function in Equation (5) with a U0.16 format.

Table 1 summarizes the mathematical representations employed for the parameters input to the scatterer generator.

### 3.3. Pipeline Implementation and Dynamics of the Calculations

**Figure 1:**
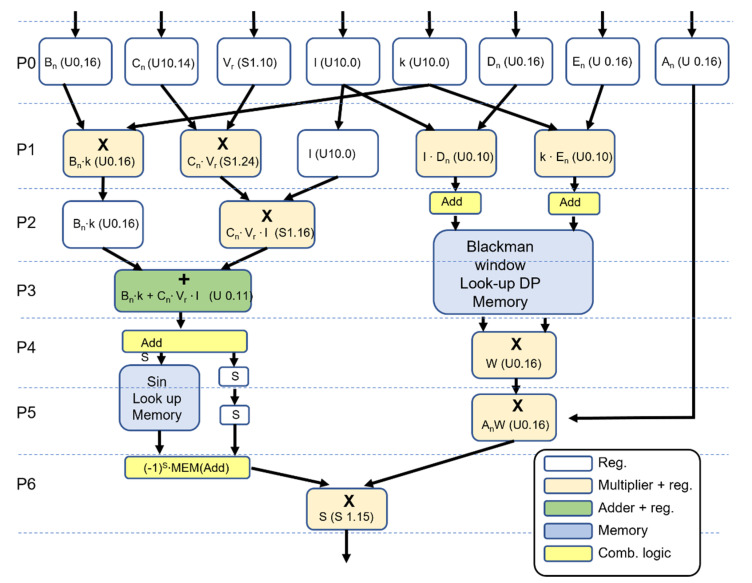
Pipeline of the scatterer generator.

The scatterer generator performs the calculation of the scatterer data according to Equation (5) in the seven-stage pipeline sketched in Figure 1. This architecture synthetizes a sample per clock cycle with a seven-cycle latency. The pipeline stages are named P0–P6. In the figure, the pink and green blocks represent synchronous multipliers and adders, respectively; the white blocks are simple registers, the yellow blocks represent combinatorial operations, and the azure blocks are synchronous memories. In particular, the memories span two pipeline stages since they feature a two clock cycle latency: in the first cycle the address is latched in the input register, in the second the corresponding data are latched in the output register.

Data enter the pipeline in the format previously described and summarized in Table 1. Throughout the processing path, data maintain the fixed-point format. However, the use of unsigned or two’s complement format, the number of bits, and the position of the fractional point change according to the specific operation. The mathematical representation employed in each of the pipeline steps is reported in Figure 1. The pipeline operations are detailed below:

P0: In the first stage data are latched in the input registers.

P1: The calculation of the argument of the sin(2π(·)) function starts with the parallel calculation of the $B_{n}\cdot k$ and $C_{n}\cdot V_{n}$ products. The $B_{n}\cdot k$ multiplication is performed at 26 bits, but the 10 MSBs are truncated for the property of Equation (7), while the fractional part is maintained with 16-bit resolution. The final format is U0.16. The $C_{n}\cdot V_{n}$ product is calculated at 35 bits, and, for the same property discussed above, the integer part is ignored. Since the result is signed, the sign bit is propagated together with the 24 bits of the fractional part, and the format is S1.24.

The calculation of the two addresses that feed the tapering window table $W_{n}\left(k\right)$ and $W_{n}\left(l\right)$ starts by calculating $l\cdot D_{n}$ and $k\cdot E_{n}$. The unsigned products are carried out at 26 bits, but only the 10 MSBs of the integer parts are saved in the format U10.0.

P2: The $C_{n}\cdot V_{n}$ value is multiplied for *l* in a signed operation. The integer part is ignored again for the property of Equation (7), and 16 bits of the fractional part proceeds to the next stage together with the sign. The two addresses for the 512 memory table for the Blackman window are calculated by applying Equation (10) to the $l\cdot D_{n}$ and $k\cdot E_{n}$ values. The results are latched in each of the two ports present in the dual port memory. Finally, the coefficient *l* is registered to maintain the data alignment in the pipeline.

P3: The argument of the sin[2π(x)] is calculated by executing the last signed summation. It should be noted that, for the application of the property of Equation (7), the sign and the integer part of the result is ignored, and 11 bits of the fractional part are obtained with a simple truncation. The final result is unsigned with the format U0.11.

The values of the Blackman window are latched in the output registers of the look-up memory.

P4: The address to the 512 look-up that stores the quadrant of the sin function is calculated by Equation (11) and latched in the memory. The sign of the sin is propagated. Simultaneously, the two values of the Blackman windows $W_{n}\left(k\right)$ and $W_{n}\left(l\right)$ in output from the dual port look-up memory are multiplied and registered in the U0.16 format.

P5: The value of the sin look-up memory is latched in the output register and the sign is propagated again. The combined tapering value is multiplied to $A_{n}$ and saved in the U0.16 format.

P6: The value of the sin function is reconstructed according to Equation (11) from the sign and the first quadrant data are read from the memory in a combinatorial logic. The output is multiplied to $A_{n}W_{n}\left(k\right)W_{n}\left(l\right)$ obtained at the previous step. The final result is registered in the S1.15 format.

### 3.4. The Scatterer Generator Co-Processor

**Figure 2:**
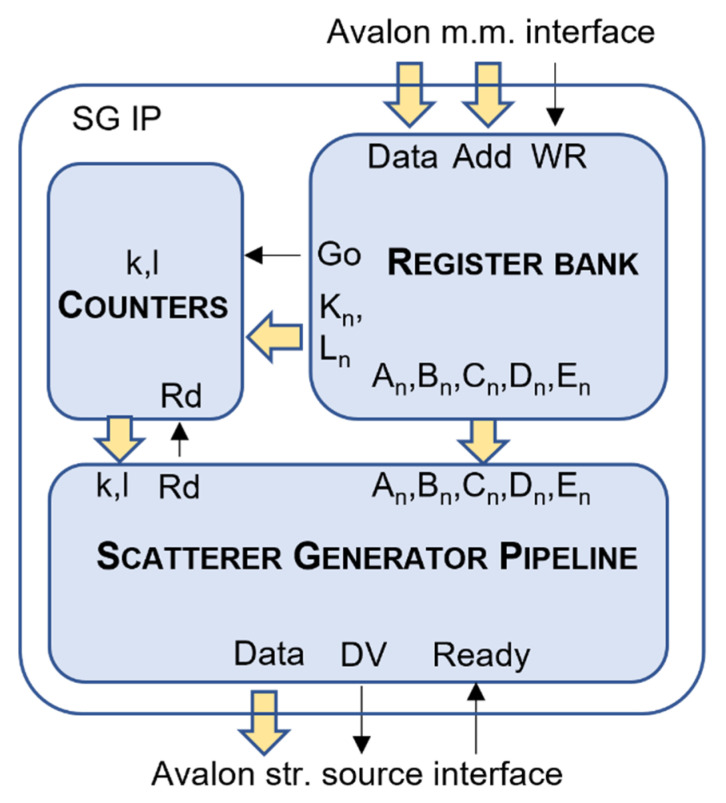
Scatterer generator integrated in the SG IP with input/output interfaces and counters for the *l*, *k* indexes.

The pipeline described in the previous section is an efficient architecture that performs the calculations required for the synthesis of the scatterer samples. In order to be exploited in a wider FPGA structure, it should be completed with input/output interfaces and a simple logic for the generation of the *l* and *k* indexes. This is done in the scatterer generator IP (SG IP) shown in Figure 2.

An Avalon memory mapped interface [26] is used to access the IP and to write the values of the input registers reported in Table 1, apart from *l* and *k*, that are generated internally. The registers are mapped in memory at the addresses described in Table 2. The interface features a 4-bit address bus, a 32-bit data bus, and the write command. The bus master writes the register values and then performs a write access at the address 8h that triggers the generation of the data.

Two counters generate the *k* and *l* indexes in the range 0, $K_{n}-1$ and 0, $L_{n}-1$, respectively. The index *k* is incremented for every clock cycle; when it reaches $K_{n}-1,$ it is zeroed and *l* is incremented. When both indexes are at their upper limit, the data generation stops.

Elaborated data are available in output through a streaming source Avalon interface [26]. Every clock cycle, a new 16-bit sample is available; Data Valid (DV) and Ready signals perform the handshake between source and sink interfaces. In particular, if the sink interface deasserts the Ready signal, the *k* and *l* counters halt and the pipeline stalls.

The SG IP represents an efficient and complete scatterer generator coprocessor that can be integrated in a FPGA project. The SG IP is freely available as Supplementary Materials attached to this paper.

### 3.5. FPGA Integration of the SG IP

The SG IP acts as a slave coprocessor and should be integrated to work in a wider processing architecture. The specific architecture depends on the final application the user is developing; however, at least the following three elements should be present:

- a processor that sets and commands the SG IP through the input memory mapped bus;
- an adder chain where the scatterer accumulations of Equation (4) are performed;
- quick access to a large memory buffer (e.g., DDR memory) where the scatterer matrix is stored.

**Figure 3:**
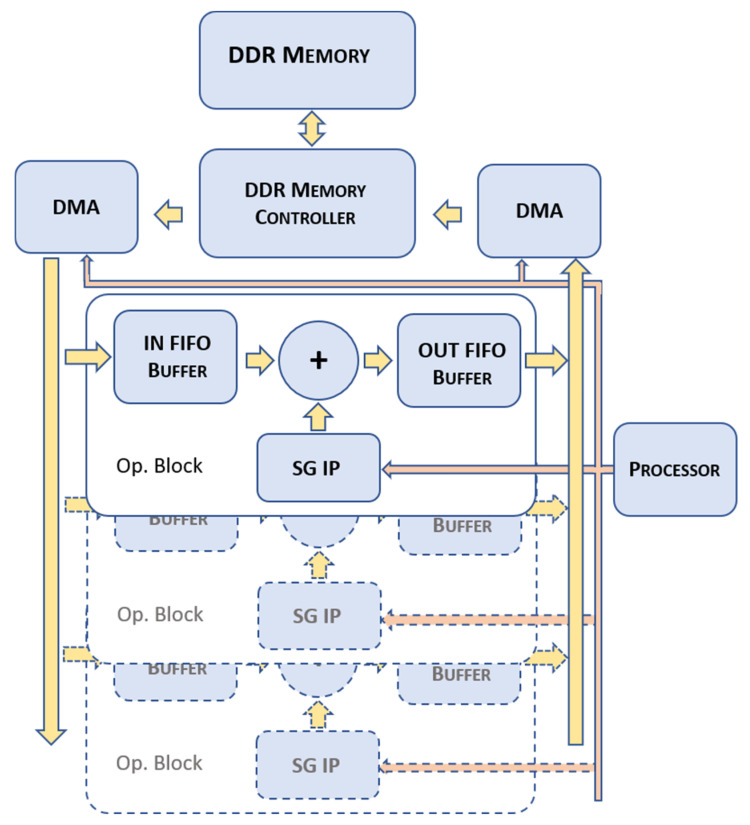
Example of architecture with a processor connected to multiple SG IPs working in parallel.

Figure 3 reports an example of a possible architecture that integrates one or more SG IPs that work in parallel. An input First-In-First-Out (IN FIFO) memory, an adder, the SG IP, and an output FIFO (OUT FIFO) constitute the operational block (Op. Block) that can be instantiated one or more times (see Figure 3). A processor, through the memory mapped bus (pink in the figure), sets the working parameters of the SG IPs and the two Direct Memory Access (DMA) blocks. The DMA blocks access the Double Data Rate (DDR) memory through the DDR controller. The left DMA block moves data from the DDR memory to the IN FIFO memories of each Op. block. Data from the IN FIFOs is added to the data generated by the SG IPs and the results feed the OUT FIFO memories. Data from the OUT FIFOs of each Op. Block are moved back into DDR memory by the right DMA block.

In this architecture, the processor coordinates the DMA blocks and the SG IPs to generate the matrix of Equation (4) in the DDR memory. A possible sequence of operations for the processor is the following. The processor generates hundreds of thousands of random positions ($K_{n}/2,L_{n}/2$) that map the scatterers in the matrix of Equation (4). Then, for each position:

- it programs the DMA to move the data corresponding to the samples to be generated into the IN FIFO of one of the Op. Blocks;
- it programs the SG IP to generate the new samples, which are added to the old data and moved into the OUT FIFO;
- it programs the DMA to move the data back to its original position in DDR.

If more than one Op. Block is instantiated, the processor manages the Op. blocks to produce data belonging to different regions of the DDR buffer in parallel.

### 3.6. Flow Emulator: An Example of SG IP Employment

This section reports an example of how the SG IP can be exploited in a practical application. In particular, the SG IP is here employed for the real-time emulation of a Doppler echo signal that can be used to test echographic equipment. Details of the embedded electronics system that includes the SG IP can be found in [21]; a brief summary is given here for reader convenience.

The architecture proposed in Figure 3 was integrated in the System on Chip (SoC) 5CSXFC6C6U23C7 FPGA of the Cyclone V family (Intel-Altera, Santa Clara, CA, USA). A single Op. Block was used. The SoC FPGA included an 800 MHz dual-core ARM processor that was programmed to manage the SG IP and the DMA blocks. The FPGA was part of the System-On-Module (SOM) MitySOM-5CSX-H6-42A-RC, produced by Critical Link, LLC (Syracuse, NY, USA). In addition to the FPGA, the module featured an Ethernet controller, two DDR memory buffers (256 MB connected to the FPGA fabric, 1 GB dedicated to the ARM processor), and other peripherals not used in this application. The system was completed by a custom board with a Digital-to-Analog (DA) converter and a front-end to drive an ultrasound transducer.

**Figure 4:**
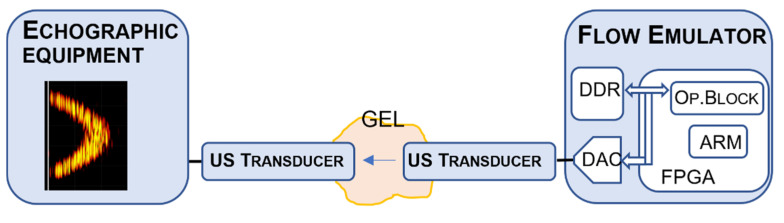
Acoustic coupling between the flow emulator based on the SG IP and an echograph system.

In this application, the ARM processor manages the architecture of Figure 3, which synthetizes the matrix of Equation (4) in the DDR memory according to the parameters set by the user. The buffer is then reproduced at the Pulse Repetition Interval (PRI) through the DA converter and the ultrasound transducer (See Figure 4). The transducer is placed in front of the probe of the echographic equipment being tested and coupled through ultrasound gel. The echograph receives the echoes emulated by the flow emulator board and processes them as if they came from a real flowing fluid.

## 4. Experiments and Results

### 4.1. Performence of the SG IP: Resources, Througput, Precision

Table 3 summarizes the FPGA resources required by the SG IP in terms of Adaptive Logic Modules (ALMs), memory bits and Digital Signal Processors (DSPs). The Table 3 reports also that the project reached the time closure working with a clock of 150 MHz.

Working with a clock of 150 MHz, each SG IP features a peak throughput of 150 M sample/s. A scatterer is composed by $K_{n}\cdot L_{n}$ samples; in order to generate a real-like flow signal, it is necessary to achieve a scatterer density of about 10 per SV. This means that every sample of the matrix of Equation (4) should be, on average, the summation of 10 overlapped scatterers. In other words, the samples of the matrix are generated at a peak rate of:

(12) $$\frac{N_{p}}{s}=\frac{150\;\mathrm{Ms}}{s}\cdot \frac{1}{D}$$

where *D* is the scatterer density. The peak rate corresponds to 15 Ms/s for *D* = 10. However, the average rate is less than this, since some time is required to set the SG IP before the generation of each sample, and the latency of the DDR memory controller can add further dead time. For example, the architecture implemented in [21] achieved $\frac{N_{p}}{s}$ = 50/*D* Ms/s, which is more than enough for real-time synthesis of the signal. For comparison, we implemented Equation (3) in Matlab (The Mathworks, Natick, MA, USA) and ran it on a PC with an Intel^®^ Core™ i7-2670QM at 2.2 GHz and 8 GB of RAM. It generated $N_{p}/s$ = 4 × 10^5^ sample/s, i.e., the embedded system was 30-fold more rapid than the PC. The algorithm can, theoretically, be implemented on a microcontroller or embedded processor. If we consider, for example, a 100 MHz microcontroller able to perform a mathematical operation in 10 clock cycles, considering that Equation (1) requires 10 operations per sample in addition to sin and Blackman window calculation, the microcontroller will calculate, at best, 1 M sample/s. This corresponds to $N_{p}/s$ = 100k per D = 10, which is far too low for real-time performance.

The scatterer generator performs the calculations in the fixed-point mathematical representation detailed in the previous paragraphs. The limited dynamics of this format produces small errors whose effects results in background white noise. In this section, the mathematical noise produced by the scatterer generator is quantified. The calculation chain implemented in the scatterer generator was implemented in FPGA and also reproduced in Matlab, where the calculations were performed in 64-bit floating-point format and considered as reference. A total of 40 matrices, corresponding to 40,000 scatterers, were generated starting from the same parameters both in the FPGA, $M_{C}\left(k,l\right)$, and in Matlab, $M_{R}\left(k,l\right)$. The matrices were then compared and the Signal-to-Noise Ratio (SNR) was calculated according to the following metrics:

(13) $$\mathrm{SNR}=10{\mathrm{Log}}_{10}\left(\frac{\sum_{k}^{}\sum_{l}^{}{\left(M_{C}\left(k,l\right)\right)}^{2}}{\sum_{k}^{}\sum_{l}^{}{\left(M_{C}\left(k,l\right)-M_{R}\left(k,l\right)\right)}^{2}}\right)$$

The SNR averaged over the 40 matrices was 61.4 dB, with a standard deviation of ±0.8 dB.

### 4.2. Experiments

The proposed coprocessor, integrated in the flow emulator system, was tested by emulating a flow for the ULA-OP research scanner [27]. The two apparatuses were acoustically coupled. A 7 MHz piston transducer with 70% bandwidth was connected to the flow emulator system and placed in front of a LA533 probe, at about 1 cm. The LA533 probe is a linear array produced by Esaote (Genova, Italy) composed by 192 elements and optimized for biomedical vascular investigations. Ultrasound gel was interposed in between the probe and the piston transducer to allow good acoustic coupling. An electrical connection was used for the PRI synchronism generated by the ULA-OP [28]. The Flow Emulator was set to emulate a Newtonian fluid (parabolic velocity profile) with a diameter of 5.5 mm and a peak velocity of 0.35 m/s. The velocity was emulated with the temporal trend typical of carotid flow, with a heart rate of 60 ppm. The echograph was set for a Doppler investigation at 7 MHz with PRF = 6 kHz. The echograph transmission was disabled. The display was set to show the spectral velocity profile [18] and the sonogram.

**Figure 5:**
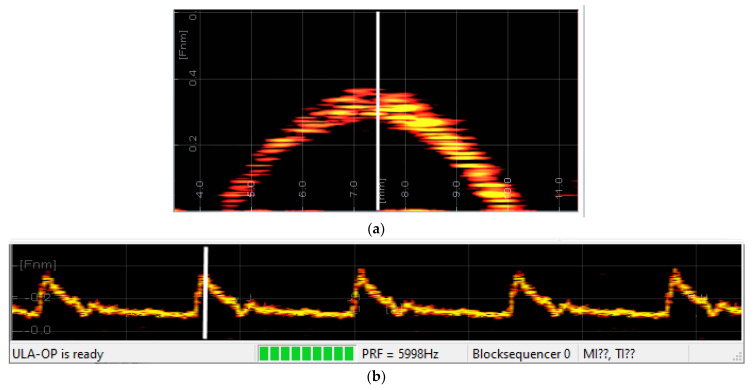
Screenshot of the real-time echograph display showing the sonogram (**a**) and the spectral profile (**b**) corresponding to the Doppler signal emulated by the proposed coprocessor. The power of the Doppler spectrum is represented in colors from red (low power) to yellow (high power). The sonogram refers to the middle-vessel depth, highlighted by the white line in the profile.

During the experiment, the echograph acquired the signal and processed it in real-time through complex demodulation [9], filtering, and 128-point FFT (packet size 128) to obtain the spectral velocity profiles and the sonogram. Figure 5 presents a screenshot of the real-time display of the ULA-OP echograph showing the result of the Doppler analysis. The (a) panel shows the spectral velocity profile frozen during the velocity peak corresponding to the white line in the sonogram. The (b) panel reports the sonogram corresponding to the center of the flow, highlighted by the white line in the velocity profile. The Doppler shift measured by the echograph at maximum velocity was about 3400 Hz, which, according to the Doppler formula (sound velocity 1480 m/s) corresponds to 3400/7,000,000 × 1480/2 ≈ 36 cm/s, which agrees well with the emulated velocity.

## 5. Discussion and Conclusions

This work was focused on a SG IP coprocessor designed to speed up the generation of a synthetic signal that emulates the echoes backscattered by a moving fluid when investigated by a Doppler system. The SG IP is a slave coprocessor and it needs a master processor to work with. The master processor can be a soft-processor, such as the Intel-Altera Nios II, or a hard processor, such as that integrated in the System-on-Chip (SoC) FPGA and employed in the presented integration, or it can be even a processor external to the FPGA.

The presented coprocessor can be employed in a parallel architecture to improve the throughput. However, employing more than one coprocessor is rarely necessary. For example, the presented integration produced a peak throughput of 150 M sample/s with just one coprocessor. On the other hand, the maximum performance is limited by the DDR memory bandwidth. For example, with a 100 MHz system clock, a single SG IP produces 100 M samples/s (neglecting the few clock cycles required to set the parameters), corresponding to a data flow of 200 MB/s. A 16-bit 400 MHz DDR memory features a peak bandwidth of 1600 MB/s. The memory can move about 800 MB/s back and forth. In this condition, the memory bandwidth limits the parallelism to no more than three to four coprocessors (Op. Blocks in Figure 3).

The SG IP coprocessor requires very few FPGA resources and thus can also be implemented in entry-level devices, allowing the realization of low-cost flow-emulator embedded systems. The architecture of the proposed IP is scalable; future work will focus on the design of multi-channel flow emulators capable of simultaneously generating different signals to be electrically coupled to multi-channels echographs. These emulators will allow accurate tests of beamforming sequences, plane wave techniques [29], and vector methods [13] that are implemented in 2D/3D imaging Doppler systems [30].

## Figures and Tables

**Table 1:** Parameters input to the scatterer generator and their mathematical representations.

$A_{n}$	$B_{n},$	$C_{n},$	$D_{n},$	$E_{n}\;$	$V_{n}\;$	$k,K_{n}\;$	$l,L_{n}$
U0.16	U0.16	U10.14	U0.16	U0.16	S1.10	U0.10	U0.10

**Table 2:** Addresses of the parameters in the input Avalon memory mapped interface.

Address	Parameter
0 h	$B_{n}$
1 h	$C_{n}$
2 h	$D_{n}$
3 h	$E_{n}$
4 h	$V_{n}$
5 h	$A_{n}$
6 h	$L_{n}$
7 h	$K_{n}$
8 h	Go

**Table 3:** Resources required by the SG IP and working frequency.

ALMs	Memory Bits	DSPs	Freq.
77	15872	8	150 MHz

## Data Availability

The FPGA code sources presented in this study are available in Supplementary Materials.

## Supplementary Materials

The following are available online at https://www.mdpi.com/article/10.3390/mi12121549/s1, FPGA source code of the coprocessor.

## Appendix A

Table A1 reports the main acronyms employed in this paper with their meaning and, when applicable, the unit.

**Table A1:** Nomenclature employed in the paper.

Acronym	Meaning	Unit
PRI	Pulse Repetition Interval	s
PRF	Pulse Repetition Frequency	Hz
FPGA	Field Programmable Gate Array	-
VHDL	VHSIC Hardware Description Language	-
SoC	System-on-Chip	-
EDP	Electronics Doppler Phantom	-
SV	Sample Volume	m
SG IP	Scatterer Generator Intellectual Property	-
DMA	Direct Memory Access	-
DDR	Double Data Rate	-
ALM	Adaptive Logic Module	-
DSP	Digital Signal Processor	-
SOM	System On Module	-
SNR	Signal-to-Noise Ratio	-
FFT	Fast Fourier Transform	-
